# Airspace closures due to reentering space objects

**DOI:** 10.1038/s41598-024-84001-2

**Published:** 2025-01-23

**Authors:** Ewan Wright, Aaron Boley, Michael Byers

**Affiliations:** 1 Interdisciplinary Studies Graduate Program, University of British Columbia, Vancouver, BC Canada; 2 Department of Physics and Astronomy, University of British Columbia, Vancouver, BC Canada; 3 Department of Political Science, University of British Columbia, Vancouver, BC Canada

**Keywords:** Sustainability, Socioeconomic scenarios, Environmental impact

## Abstract

Uncontrolled reentries of space objects create a collision risk with aircraft in flight. While the probability of a strike is low, the consequences could be catastrophic. Moreover, the risk is rising due to increases in both reentries and flights. In response, national authorities may choose to preemptively close airspace during reentry events; some have already done so. We determine the probability for a rocket body reentry within airspace over a range of air traffic densities. The highest-density regions, around major airports, have a 0.8% chance per year of being affected by an uncontrolled reentry. This rate rises to 26% for larger but still busy areas of airspace, such as that found in the northeastern United States, northern Europe, or around major cities in the Asia-Pacific region. For a given reentry, the collision risk in the underlying airspace increases with the air traffic density. However, the economic consequences of flight delays also increase should that airspace be closed. This situation puts national authorities in a dilemma—to close airspace or not—with safety and economic implications either way. The collision risk could be mitigated if controlled reentries into the ocean were required for all missions. However, over 2300 rocket bodies are already in orbit and will eventually reenter in an uncontrolled manner. Airspace authorities will face the challenge of uncontrolled reentries for decades to come.

## Introduction

This paper builds on a preliminary study presented by the authors at the 2nd International Orbital Debris Conference^1^. While there is overlap in the text, this article greatly expands upon that initial work.

On 4 November 2022, a 20 tonne Long March 5B (LM-5B) rocket body reentered the atmosphere over the Pacific Ocean. This reentry location was a result of chance rather than design, as the rocket body was abandoned in orbit and left to return to Earth in an uncontrollable manner. In the evening before its reentry, the rocket body was predicted to reenter over southern Europe. This led the European Union Space Surveillance and Tracking organisation (EU SST) to raise concerns with the European Aviation Safety Agency (EASA), which in response issued a Safety Information Bulletin (SIB) recommending that national authorities ‘consider implementing and notifying airspace restrictions on a path of minimum 70 km and up to 120 km on each side of the estimated re-entry trajectory’^2^. The following morning, Spanish and French authorities elected to do just that, and closed part of their airspace.

**Fig. 1:**
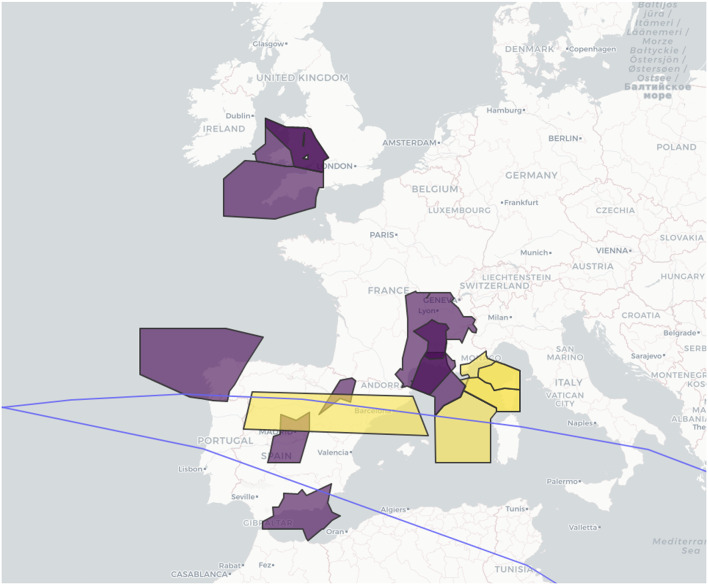
Long March 5B reentry tracks (blue line) over Europe on 4 November 2022 with zero rate (closure) NOTAMs in yellow and related non-zero rate (advisory) NOTAMs in purple.

Of the 15 notices to air missions (NOTAMs) issued across Europe for the reentry, four were zero-rated, meaning the airspace was effectively closed (Fig. 1). These closures delayed 645 aircraft by an average of 29 min, and covered central Spain, southern France, and Monaco^3^. Particularly disruptive were the short lead times between the announcement of a closure and its implementation—in some cases, aircraft already in flight had to be diverted or instructed to return to their departure airport. Surrounding states, including Portugal, Italy and Greece, were also under the reentry flight path and subject to the same reentry risk, but elected not to close their airspace. They then saw an unexpected increase in air traffic from diverted flights, which carried different, operational risks. The incident highlighted, among other things, a lack of preparation for this eventuality and a lack of harmonization of responses among states.

The final orbit track is the upper of the two paths—taken before the rocket body deorbited in the Pacific. Had it not deorbited, it would have followed the lower track approximately 1.5 h after the first pass. Image from EUROCONTROL^3^. Note the designations employed do not imply the expression of any opinion whatsoever on the part of EUROCONTROL concerning the legal status of any country, territory, city, or area or of its authorities, or concerning the delimitation of its frontiers or boundaries.

This was the first time that airspace was closed due to an uncontrolled reentry, though warning NOTAMs have been issued for numerous reentries, as have EASA SIBs^4,5^.

The aggregate probability of reentry debris striking an aircraft is small. However, since an aircraft strike can lead to mass casualties, the risk arising from such an event is of real and growing concern. The number of daily flights has almost doubled since 2000, with the number of passengers increasing by over 250%^6,7^. The trackable objects in orbit have more than doubled in the last decade^8^, and large reentries occur almost weekly^9^. There were a record 212 successful launches in 2023, from which 128 rocket bodies were abandoned in orbit to reenter uncontrolled^10^.

Uncontrolled satellite reentries are also risky, but rocket bodies present a particular hazard as they are often large and less likely to burn up entirely. And since many rocket bodies remain in orbit for years before reentering, there has been an accumulation of these dangerous objects: as of June 2024, there are more than 2300 of them in orbit, destined to eventually return to Earth^11^.

There are numerous instances of aircraft being struck by objects while flying at high altitude, though attribution to reentry debris is difficult^12^. Even small debris poses an acute risk because the speed of the aircraft contributes to the total kinetic energy of the collision—the speed of the aircraft is much faster than that of the falling debris^13^. Estimates suggest that debris as small as one gram could damage an aircraft, particularly if it strikes a windshield or is ingested by an engine^14^. Nine-gram steel cubes have been shown to perforate aircraft fuselages^15^, and debris with mass greater than 300 g could result in a catastrophic incident, i.e., total aircraft loss^16^.

A 2021 analysis by The Aerospace Corporation found the probability of a fatal aircraft collision with reentry debris to be $8\times {10}^{-6}$ for that year, i.e., almost 1 in 100,000. When the reentries from twelve future megaconstellations were included, this probability was predicted to rise to $7\times {10}^{-4}$ (7 in 10,000 per year) in 2035. Because each aircraft carries many passengers, the 2021 annual risk of one or more casualties from debris-aircraft collisions was estimated to be 0.1%, rising to 0.84% by 2035^17^.

These are aggregate probabilities involving many future reentries, but the risks from individual reentries may rise to a level where a safety concern is present. For example, though not an uncontrolled reentry, the 2003 Space Shuttle Columbia disaster saw numerous flights continuing under the falling debris from the exploded spacecraft. Distributed over hundreds of kilometers, the debris fell for a surprisingly long time: most of the debris fell within 40 min, with confetti-like pieces lingering for up to 2 h. Post-event analyses estimated that the risk to aircraft in the area was 0.3–10%^18^.

In the United Kingdom, the National Space Operations Centre monitors daily uncontrolled reentries^19^, the Civil Aviation Authority is notified if the risk of a reentry in UK airspace is above 1%, and an airspace closure considered if the risk is above 5%^20^. Yet safety-oriented actions, such as precautionary airspace closures, have their own consequences. One is the operational risk of diverting many aircraft; another is the economic impact.

Each delayed flight accrues numerous costs, including costs to airlines (staff costs and knock-on scheduling issues), costs to passengers (value-of-time costs), and wider economic impacts. Precautionary airspace closures from reentries have been estimated to cost potentially up to tens of millions of dollars^21^. In the case of the November 2022 LM-5B reentry, this is a cost that was paid by the airlines and their passengers. But it was created by the space industry. The space industry is thus exporting negative externalities onto the aviation industry. As we have explained elsewhere, the “launch state” could be liable under international law, not just for any physical damage that might occur, but for the economic damage of a precautionary airspace closure^3^.

Airspace closures are a blunt mechanism to reduce risk, but national authorities may decide this is preferrable to other options, particularly when reentry risks are hard to quantify, as we discuss below. The November 2022 airspace closure is unlikely to be the last. Here, we model probabilities for reentries over regions with high air traffic densities, as well as the resulting risks to aircraft in flight, based on flight data from 2023. We then discuss the possible consequences of preventive actions and the difficulties this creates for policymakers.

## Results

### Aircraft distribution

**Fig. 2:**
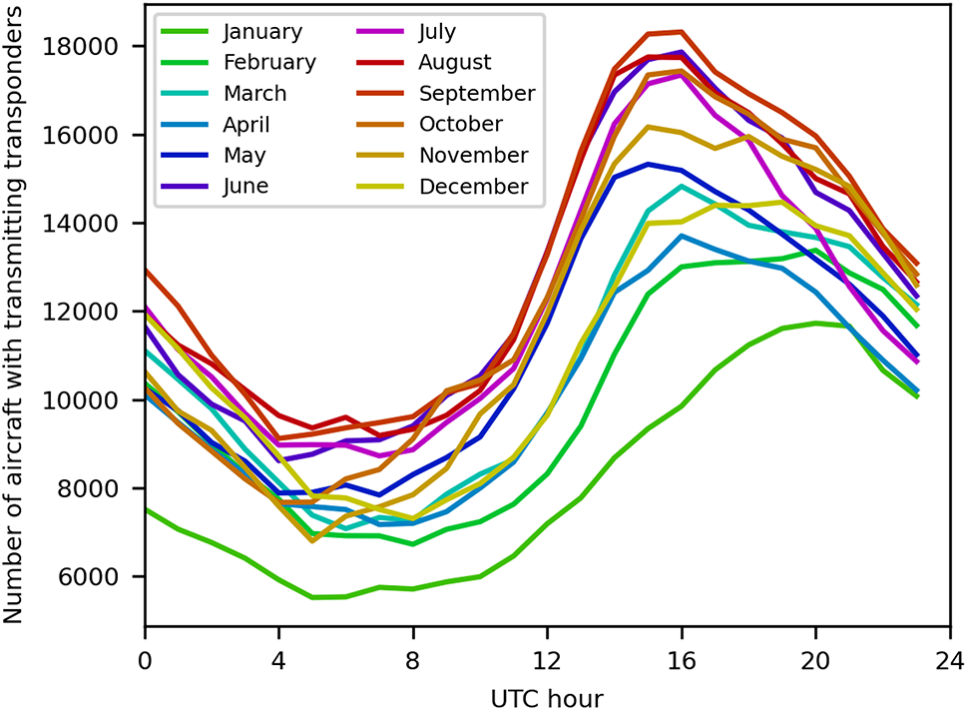
Number of aircraft in the sky throughout the first day of each month in 2023, sampled hourly. In general, the number of aircraft peaks in the Northern Hemisphere summer, though other periodic effects such as the day of the week and year (e.g., New Year’s Day) will also affect the number.

Using Automatic Dependent Surveillance (ADS) transponder data used to track aircraft, we modelled the global distribution of aircraft in 2023 to find regions of high-density air traffic. Here, air traffic density is a column density representing the number of aircraft in the air per km^2^ above a given location. Figure 2 shows the hourly number of aircraft transmitting location information throughout the first day of each month in 2023. The date of 1 September 2023 had the maximum number of transmitters in this sample, at 18,318 at 1600 UTC. The peak number of aircraft transmitting for any area is generally found in the late afternoon local time, falling to a minimum at night. On average, 13,197 aircraft were transmitting transponder signals at any given time during this 24-h period. Since flight volumes are expected to increase in 2024 and beyond, we use 1 September 2023 for our models^22^, hereafter DS23.

Over 500 aircraft types were found in DS23. Some of the aircraft are small general aviation aircraft which have a lower area exposed to reentry debris than larger commercial aircraft; for example, there were 950 Cessna 172s at 1600 UTC. However, of the top 10 most common aircraft, eight were commercial airliners, including 1421 Boeing 737s, 1037 Airbus A320s and 571 Airbus A321s.

### Casualty expectation

Since each aircraft is moving at considerable speed, the total ‘exposed area’ to debris is larger than the actual surface area of the aircraft. We assume an average exposed area for all aircraft (see “Methods”), and that the uncertainties in other aspects of this modelling will outweigh this simplification.

By multiplying the air traffic density at each hour with the rocket body weighting function (scaled for 1 h) and the exposed area, we obtain an average hourly collision expectation for DS23 of $2.6\times {10}^{-10}$ and a total day collision expectation of $6.4\times {10}^{-9}$. If every day in 2023 had the same flight distribution as September 1, the year’s collision expectation would be $2.3\times {10}^{-6}$. This corresponds to an annual probability of a collision between a rocket body and an aircraft of $2.3\times {10}^{-6}$ (1 in 430,000) in 2023. This number is conservative because it assumes that the rocket body remains in one piece; i.e., just one piece of debris per reentry. This number is also lower than The Aerospace Corporation estimate for 2021 discussed above, but that calculation included reentering satellites and multiple debris pieces per object^17^. Assuming 200 people per aircraft, the corresponding 2023 casualty risk due to reentry debris from rocket bodies striking aircraft is $4.6\times {10}^{-4}$, or 1 in 2200.

### High air traffic density effects

**Fig. 3:**
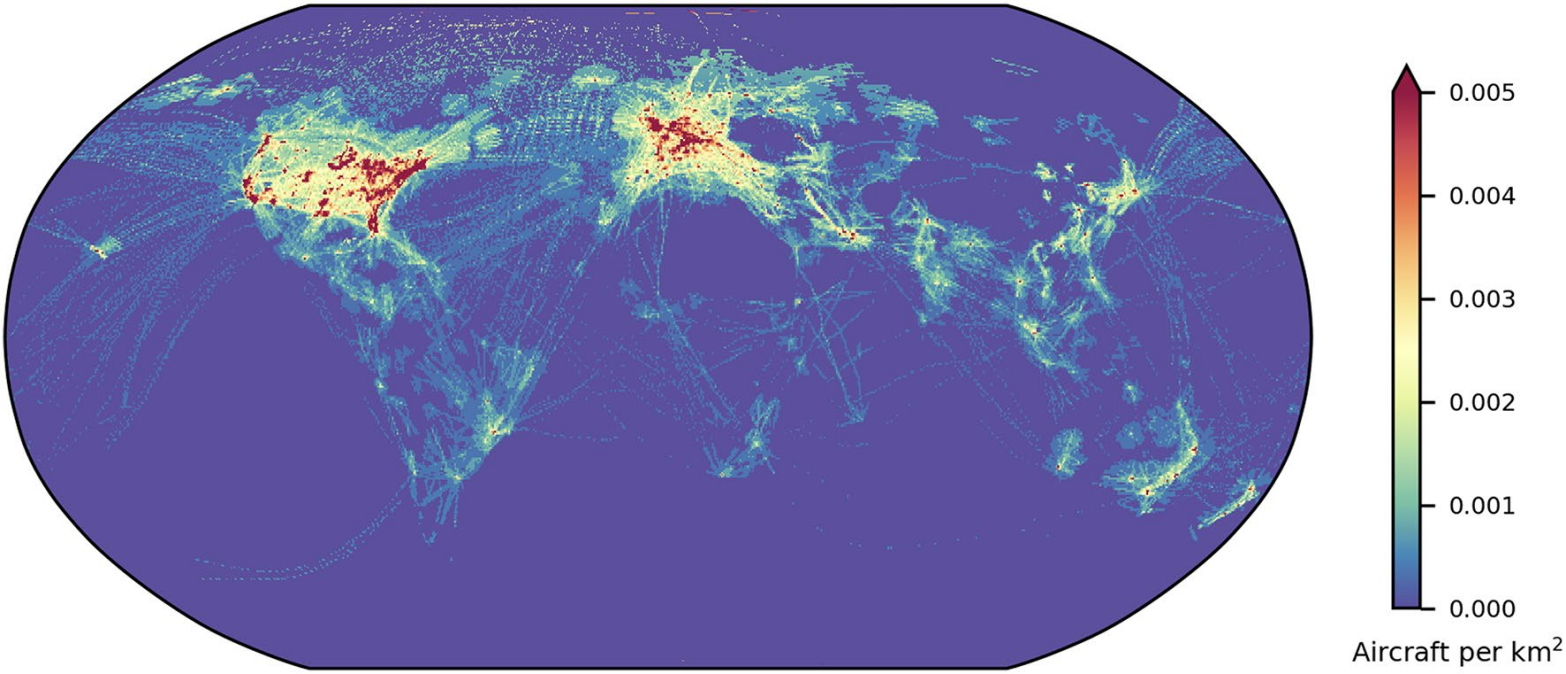
Maximum air traffic density (# km^−2^) within each 0.5° by 0.5° grid cell during 1 September 2023, for the whole world. Plotted using Cartopy v0.22.1 (scitools.org.uk/cartopy).

**Fig. 4:**
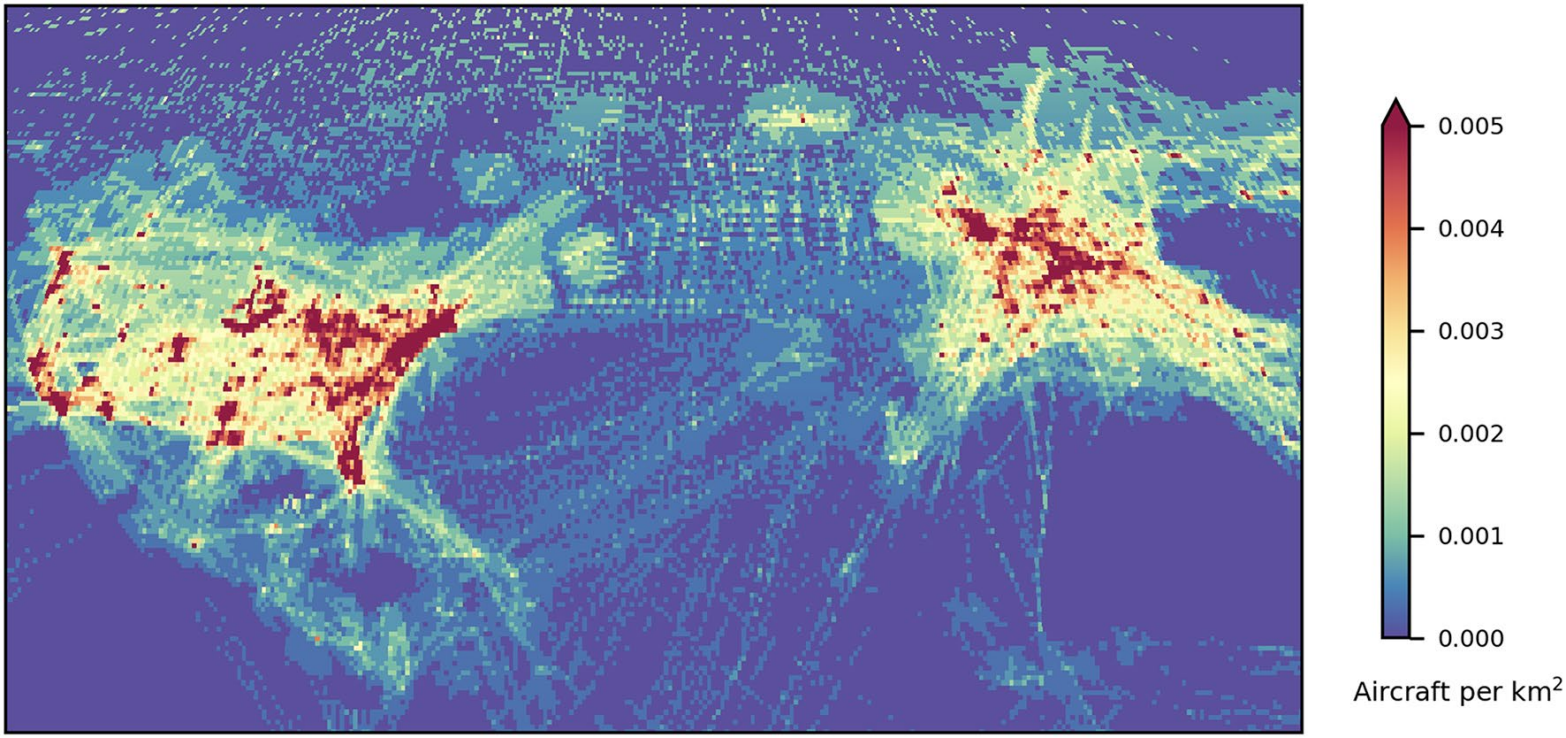
Maximum air traffic density (# km^−2^) within each 0.5° by 0.5° grid cell during 1 September 2023, for North America, the Atlantic and Europe. Plotted using Cartopy v0.22.1 (scitools.org.uk/cartopy).

The above is a model for the collision risk to aircraft. The risk of a reentry disrupting aircraft operations is different altogether. We model this as the probability that a reentry will occur over a given air traffic density threshold, as outlined in the “Methods” section. Firstly, Figs. 3 and 4 show the maximum air traffic density found in each grid cell for DS23, with aircraft distribution samples taken every 10 s.

Airspace with particularly dense air traffic is found in the United States, Europe, and the Asia-Pacific, including Japan, eastern China and Australia. The maximum air traffic density of aircraft transmitters in DS23 was 0.054 per km^2^, observed around Denver, Colorado. This is on average one aircraft every 18 km^2^. Because modelling air traffic densities can be complex, and there is no clear definition for high-density traffic, we use this peak density as a reference for various thresholds.

**Fig. 5:**
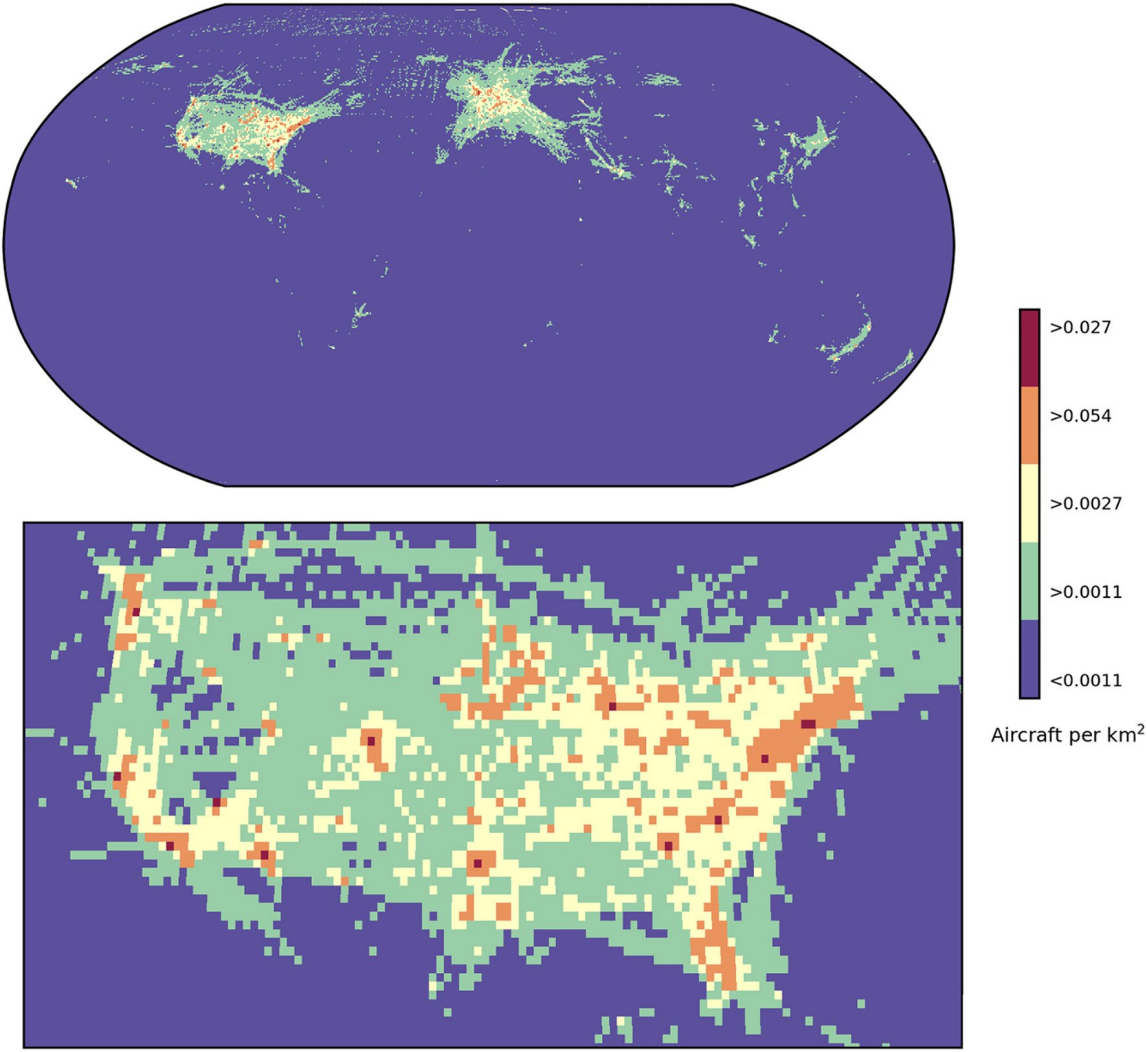
Maximum air traffic density for each grid square seen on September 1, 2023, separated by air traffic density thresholds, for the whole world and zoomed for the US. Each year across the world there is a 99% chance that rocket body reentries will occur in the green areas, 75% chance in the yellow areas, 26% chance in the orange areas and 0.8% chance in the red areas (which correspond to major airports around the world). Plotted using Cartopy v0.22.1 (scitools.org.uk/cartopy).

Considering densities that are 50% of the peak or greater (peak-50) selects only regions that contain major airports. The associated annual probability of a rocket body reentering through these relatively small regions is 0.8%, as shown in Table 1; Fig. 5. Lowering the threshold to peak-10 (10% of the peak or greater) naturally includes a much larger portion of the globe but is still focused on only busy regions around airports, such as the northeastern United States and northern Europe. At this threshold, the annual reentry probability is 26%.

Notably, the airspace over southern Europe that was closed on 4 November 2022 is only peak-5—around the world, there is a 75% chance of a reentry in such regions each year. Again, as the threshold is decreased, larger regions of the globe are included, with peak-5 enclosing much more of the Asia-Pacific region and the US (see Fig. 5). However, peak-5 remains a relatively small fraction of the globe overall, with the high chance of a reentry within that threshold reflecting the many reentries in a given year.

**Table 1:** The annual probability of a reentry occurring in several densities of airspace.

% of peak air traffic density	Average air traffic density threshold		Annual probability of infringement
	Aircraft per km^2^	km^2^ per aircraft	
50	0.027	37	0.8%
10	0.0054	180	26%
5	0.0027	370	75%
2	0.0011	920	99%

## Discussion

The consequences of a collision between an uncontrolled reentering rocket body and an aircraft would be devastating, not just to the people on board, but to the space and aviation industries, as well as the global population that depends on their services.

To reiterate, the number of launches each year and the number of rocket bodies abandoned in orbit are growing, as is the number of flights. The increase in launches is also connected to the deployment of satellite megaconstellations of thousands of satellites, such as SpaceX’s Starlink, which will in turn lead to many uncontrolled reentries of satellites. As they are unlikely to be able to conduct controlled reentries, if these or other satellites do not burn up entirely, then they too will add a significant factor to the actual collision risk^17^.

As for airspace disruptions, there is an approximately 26% probability that a rocket body reentry will occur in busy (peak-10) airspace each year, and a 75% chance that a reentry will occur in airspace that is as busy (peak-5) as that closed by French and Spanish regulators in response to a possible rocket body reentry in November 2022. These probabilities of disruption are also increasing.

There are multiple factors that make estimating the risk for any specific reentry difficult. Reentry location predictions have large uncertainties. The US Federal Aviation Administration (FAA) notes that ‘Even 60 min prior to reentry, the potential area over which debris may fall is still over 2000 km long’^17^. Even if we knew exactly where the reentry would occur, we would not know how the specific space objects might break up or what the resulting debris distribution would be. What we do know is that even a small piece of debris could be devastating to an aircraft. In addition, unlike the on-ground casualty risk, neither the reentry risk to aircraft nor the probability of air traffic disruptions in general are included in most pre-launch analyses.

On-ground casualty risks are commonly evaluated against the guideline of a 1 in 10,000 risk threshold^23^ but setting and applying a comparable threshold for aviation will be difficult. The specific risk will be affected by factors such as the growth in flights, the actual exposed area of aircraft, and the local time of day of reentry (which is not known in advance). Models based on annual averages will do little to reassure national authorities that any given reentry in their airspace does not pose a risk.

In some cases, due to the above uncertainties, national authorities may preemptively close airspace until there is no longer a reentry risk, as occurred in November 2022. In other cases, national authorities might choose to leave the airspace open, based on the low aggregate probability of a collision.

Otherwise, authorities may aim to balance the collision risk against the economic costs of an airspace closure. But both the risk and the economic costs scale with the number of aircraft in the area at that time. Nevertheless, authorities will be faced with difficult choices in even low air traffic density regions. There is also no obviously ‘right’ way to approach these choices, which may raise legal and ethical considerations in addition to those of safety and economics.

All of this leads us to ask: Why should authorities have to make these decisions in the first place?

Uncontrolled rocket body reentries are a design choice, not a necessity. With engines that can reignite and improved mission designs, operators can conduct controlled reentries, directing the rocket body into a remote area of the ocean away from people and aircraft. But currently, fewer than 35% of launches conduct controlled rocket body reentries^8^. If controlled reentries were used by all operators, the risks to people and aircraft would be greatly reduced. This would also reduce the risk of on-orbit collisions, with their contribution to the crisis of space debris. While US authorities have recently proposed rules to increase the use of controlled reentries, these remain to be adopted and implemented^24^.

Currently, there are more than 2300 rocket bodies in orbit, with this number increasing by 30–40 each year^25^. Nearly all of these will eventually return to Earth, on time scales that range from months to centuries. This means that, while a switch to controlled reentries will reduce risks, national authorities will still have to plan for uncontrolled reentries in their airspaces.

The aviation industry is aware of the risks from uncontrolled reentries. The issue has been raised by the Air Line Pilots Association, International^26^, the FAA^17^ and EUROCONTROL, the European airspace coordination organization^27^. The Secretary General of the International Civil Aviation Organization highlighted the growing risks at a February 2023 workshop hosted by the Outer Space Institute, McGill Institute of Air and Space Law, and International Association for the Advancement of Space Safety^12^. The workshop participants, including engineers, pilots, air traffic controllers, reentry experts, lawyers, and space policy experts, adopted the Montreal Recommendations on Aviation Safety and Uncontrolled Space Object Reentries, which are available at^28^.

Through its continued use of uncontrolled reentries, the space industry is imposing risks and costs on the aviation industry, air crews and passengers. Policy and legal changes are needed now, before a terrible accident occurs, and before more disruption results from sudden airspace closures.

## Methods

### Risk to aircraft

To calculate the risk of an aircraft strike from space debris, we first model the reentry and aircraft distributions. We then overlay these two distributions to find the collision expectation, analogous to the on-ground casualty risk. This is the same general method used in^13^ and^29^ and is outlined here.

When viewed in aggregate, space object reentries are equally likely to occur at any longitude, though the latitudinal distribution will be defined by the space object’s orbital inclination. An object with inclination 90° will be equally likely to reenter at any latitude, and an object with an inclination of 0° will certainly reenter over the equator. All other inclinations will result in the space object reentering between northern and southern latitudes corresponding to its inclination value, creating a U-shaped function for the latitude-dependent reentry probability density (see, e.g^13,30^).

If we sum the resulting probability density functions for each reentering space object, we build a weighting function showing on average the latitudes where reentries are most likely to occur. If we propagate this over all longitudes, we can work out the likelihood of each area on Earth having a space object reenter over it. This method was used in our previous work estimating the on-ground casualty risk from reentering rocket bodies^23,30^.

To determine the weighting function, we use the uncontrolled rocket body reentries from the past decade (March 2014–March 2024) as a proxy for the current reentry distribution. We only consider rocket body reentries because they present some of the largest reentry events and are less likely to demise entirely in the atmosphere. However, satellite megaconstellations could create considerable additional risks, provided that they too do not demise entirely, as highlighted in a September 2023 FAA report^17^. We use rocket body reentry data from the General Catalogue of Artificial Space Objects, finding that 528 upper stages and 482 rocket components have reentered uncontrolled since March 2014: almost two rocket bodies per week^31^.

In any given airspace, a certain number of aircraft will be flying at any time. Viewed from above, this will correspond to an air traffic density (i.e., number of aircraft per unit area). Furthermore, each aircraft will itself have a collisional area that could come into contact with reentering debris. If the aircraft were stationary, this would be its top surface area. However, both objects are moving and the effective exposed area is larger. According to^13^, a Boeing 737 has an exposed area of just under 3000 m^2^, and a Boeing 747 has an exposed area of over 9000 m^2^. The FAA report assumed an average area of 1000 m^2^, and we too use this value^17^. This area is assumed to be much larger than the area of the reentering space object and so we neglect the rocket body area, thus assuming that a rocket body is a single falling object. This is very conservative, since any rocket body will likely break into many pieces upon reentry and those pieces would be spread over a wide area, potentially hundreds of kilometres long. We do not include such spread in our model.

By multiplying the rocket body weighting function with the air traffic density and the average exposed area of an aircraft, we determine the collision expectation (E) of an aircraft strike per event, $E= W\, N\, \sigma$, where W is the weighting function, N is the air traffic density and $\sigma$ is the aircraft effective exposed area. Recall that air traffic density is used here as a column density, representing the number of aircraft in the air per km^2^ over a given location. For the collision risk calculations, the air traffic density was sampled hourly and integrated with the same weighting function. The calculation is done over one day (DS23), which is then used for estimating the yearly risk in the context of increasing annual flights. Assuming events are independent and hence following Poisson distributions, we can derive a probability of one or more aircraft collisions (${P}_{>}$) of ${P}_{>}=1-\text{e}\text{x}\text{p}\left(-q\, E\right)$, where q is the number of independent events, as well as a casualty risk (R) of R = *E q n*. where n is the assumed number of casualties per event.

### Risk to airspace

Airspace itself is complex, comprising many different categories of controlled and uncontrolled airspace which vary around the world. It is divided into many zones, such as flight information regions and terminal control areas. The boundaries of these zones often extend beyond geographical borders. We do not attempt to model airspace itself here. Rather, we seek to characterize possible air traffic densities so that we can determine probabilities that a rocket body will reenter within given thresholds.

We approach this problem by dividing the world into a 0.5° latitude by 0.5° longitude grid. We then find the maximum air traffic density for each grid cell at 10 s intervals on 1 September 2023 using aircraft transponder data. After selecting several density thresholds based on the overall maximum air traffic density, we create binary maps for various thresholds, whereby cells with maximum densities above the threshold are 1 and all other cells are 0. The reentry weighting function is then multiplied by this threshold mask and summed to give the expectation E for a reentry above the given threshold. The probability P of one or more reentries occurring within regions above the threshold is $\text{P}=1-\text{e}\text{x}\text{p}\left(-E\right)$. A reentry may not occur at the time of maximum air traffic density, but these higher density areas are likely to be managed airspaces where the reentry will still be of concern, and this factor could dominate the decision-making.

### Aircraft data

The number of aircraft in the sky at any one time varies periodically, such as by time of day and day of the year, as well as non-periodically. Aircraft traffic has grown over the past few decades—peaking in 2019 before reducing sharply through the COVID-19 pandemic^6^. By the end of 2023 it had recovered to the 2019 level and continues to grow^22^.

We use aircraft position data created by transponders fitted to nearly all commercial aircraft and made available by ADSBexchange^32^. Free sample data are provided for the first day of every month. Of all the sample days since January 2019, the day of 1 September 2023 had the most aircraft transponder transmissions at any one time. The peak number of ADSBexchange transponder readings on this day when sampled hourly was 18,318 at 1600 UTC. However, only 14,430 of these readings contained position coordinates (ADS-B and ADS-C). The remaining were other broadcast types, including over 1200 Mode-S transponders transmissions, which do not transmit position data and are generally used on smaller aircraft. We thus use the positional data for 1 September 2023 sampled every 10 s, while noting that there are variations in aircraft flight volumes, and our sample is biased by the transponder data available.

## Data Availability

Data and code for this study is available at http://github.com/etwright1/AirspaceDebris.

## References

[1] Wright, E., Boley, A. & Byers, M. Uncontrolled reentries will disrupt airspace again. In* 2nd International Orbital Debris Conference*, 6060 (2023).

[2] European Aviation Safety Agency. *Safety Information Bulletin 2022-09* (2022). https://ad.easa.europa.eu/ad/2022-09 [Accessed 10 July 2024].

[3] Hook, C., Wright, E., Byers, M. & Boley, A. Uncontrolled reentries of space objects and aviation safety. *Acta Astronaut.* **22**, 69–80 (2024).

[4] Gini, A. *FAA Issues Notam for UARS Space Debris*. Space Safety Magazine (2011). https://www.spacesafetymagazine.com/space-debris/falling-satellite/faa-issues-notam-for-uars-space-debris/ [Accessed 10 July 2024].

[5] European Aviation Safety Agency. *Safety Information Bulletin 2022-07* (2022). https://ad.easa.europa.eu/ad/2022-07 [Accessed 10 July 2024].

[6] Statista *Number of flights performed by the global airline industry from 2004 to 2021, with forecasts until 2023* (2023). https://www.statista.com/statistics/564769/airline-industry-number-of-flights/ [Accessed 10 July 2024].

[7] International Energy Agency. *World air passenger traffic evolution, 1980–2020* (2020). https://www.iea.org/data-and-statistics/charts/world-air-passenger-traffic-evolution-1980-2020 [Accessed 10 July 2024].

[8] European Space Agency. ESA’s Annual Space Environment Report (2023).

[9] Pardini, C. & Anselmo, L. The kinetic casualty risk of uncontrolled re-entries before and after the transition to small satellites and mega-constellations. *J. Space Saf. Eng.* **9**(3), 414–426. 10.1016/j.jsse.2022.04.003 (2022).

[10] McDowell, J. *Space Activities in 2023* (2024). https://planet4589.org/space/papers/space22.pdf [Accessed 10 July 2024].

[11] Celestrak.org. *SATCAT Raw satcat data* (2024). https://celestrak.org/satcat/boxscore.php [Accessed 10 July 2024].

[12] International Civil Aviation Organisation. Remarks by the Secretary General of the ICAO to the joint workshop on the risks to airplanes in flight from re-entering space debris. Available online at: (2023). https://www.icao.int/secretariat/SecretaryGeneral/Documents/Addresses%20and%20Messages/20230217_SG-SPEECH-SpaceDerbisWorkshop.pdf [Accessed 10 July 2024].

[13] Patera, R. Risk to commercial aircraft from reentering space debris. *AIAA Atmos. Flight Mech. Conf. Exhibit.*10.2514/6.2008-6891 (2008).

[14] Range Commanders Council. *Common Risk Criteria for National Test Ranges: Inert Debris*. National Technical Reports Library, NM 88002-5110 (2000).

[15] Wilde, P. *Impact Testing and Improvements in Aircraft Vulnerability Modeling for Range Safety, 7th IAASS Conference*, Germany (2014).

[16] Range Commanders Council. *Common Risk Criteria Standards for National Test Ranges* (Supplement, RCC 321-20, 2020).

[17] Federal Aviation Administration. Report to Congress: Risk Associated with Reentry Disposal of Satellites from Proposed Large Constellations in Low Earth Orbit (2023).

[18] International Association for the Advancement of Space Safety. *Making Space Safe and Sustainable* (2021). https://www.iaass.org/wp-content/uploads/2023/10/Making-Space-Safe-and-Sustainable-A4-v1-3.pdf [Accessed 10 July 2024].

[19] National Space Operations Centre & UK Space Agency. *How we protected the UK in May 2024* (2024). https://www.gov.uk/government/publications/how-we-protected-the-uk-and-space-in-may-2024 [Accessed 10 July 2024].

[20] UK Department for Transport. Informing UK launch insurance policy, and assessing risk to civil aviation from re-entry debris arising from UK spacecraft and launch vehicle debris (2023). https://www.contractsfinder.service.gov.uk/Notice/665bbbf5-b4dc-48ce-8ee1-d121d301c267 [Accessed 10 July 2024].

[21] Emanuelli, M. & Lips, T. *Risk to Aircraft from Space Vehicle Debris*. UN COPUOS Scientific and Technical Subcommittee presentation (2015). https://www.unoosa.org/pdf/pres/stsc2015/tech-29E.pdf [Accessed 10 July 2024].

[22] International Civil Aviation Organization. *Passenger air traffic surpasses pre-pandemic levels* (2024). https://www.icao.int/Newsroom/Pages/Passenger-air-traffic-surpasses-pre-pandemic-levels.aspx [Accessed 10 July 2024].

[23] Byers, M., Wright, E., Boley, A. & Byers, C. Unnecessary risks created by uncontrolled rocket reentries. *Nat. Astron.* **6**, 1093–1097 (2022).

[24] Federal Aviation Administration. *FAA Proposed Rule Would Reduce the Growth of Debris from Commercial Space Vehicles* (2023). https://www.faa.gov/newsroom/faa-proposed-rule-would-reduce-growth-debris-commercial-space-vehicles [Accessed 10 July 2024].

[25] Wright, E., Boley, A. & Byers, M. *Rocket Body Reentry Trends*. ESA Clean Space Industry Days 2023 (2023). https://indico.esa.int/event/450/contributions/8880/ [Accessed 10 July 2024].

[26] Air Line Pilots Association, International. *Letter to Secretary-General of the International Civil Aviation Organization* (2021). https://www.alpa.org/-/media/ALPA/Files/pdfs/news-events/letters/2021/0514-icao-fang-liu-rocket.pdf?la=en [Accessed 10 July 2024].

[27] EUROCONTROL. Stakeholder forum on the real risk posed by uncontrolled space object reentries to aviation. 2 May 2023 webinar (2023). https://www.eurocontrol.int/event/eurocontrol-stakeholder-forum-real-risk-posed-uncontrolled-space-object-re-entries-aviation [Accessed 10 July 2024].

[28] Outer Space Institute. *Montreal Recommendations on Aviation Safety and Uncontrolled Space Object Reentries* (2023). https://outerspaceinstitute.ca/osisite/wp-content/uploads/Montreal-Recommendations-on-Aviation-Safety-and-Uncontrolled-Space-Object-Reentries.pdf [Accessed 10 July 2024].

[29] Ailor, W. H. *Large Constellation Disposal Hazards* (The Aerospace Corporation, 2020).

[30] Wright, E., Boley, A. & Byers, M. Improving casualty risk estimates for uncontrolled rocket body reentries. *J. Space Saf. Eng.* **11**, 74–79 (2023).

[31] McDowell, J. *GCAT: General Catalogue of Artificial Space Objects* (2023). https://planet4589.org/space/gcat/ [Accessed 14 March 2024].

[32] ADS-B Exchange . *Historical Data* (2024). https://www.adsbexchange.com/products/historical-data/ [Accessed 11 Mar 2024].

